# Evaluation of Type I Interferon Treatment in Hospitalized COVID-19 Patients: A Retrospective Cohort Study

**DOI:** 10.3390/pathogens13070539

**Published:** 2024-06-26

**Authors:** Vivian Y. Tat, Pinghan Huang, Kamil Khanipov, Nathan Y. Tat, Chien-Te Kent Tseng, George Golovko

**Affiliations:** 1 Department of Pathology, The University of Texas Medical Branch, Galveston, TX 77555, USA; vitat@utmb.edu; 2 Department of Microbiology & Immunology, The University of Texas Medical Branch, Galveston, TX 77555, USA; 3 Department of Pharmacology & Toxicology, The University of Texas Medical Branch, Galveston, TX 77555, USA; kakhanip@utmb.edu; 4 Taking Our Best Shot, Galveston, TX 77555, USA

**Keywords:** COVID-19, interferons, treatments, sequelae

## Abstract

Coronavirus disease 2019 (COVID-19) continues to cause morbidity and mortality worldwide; therefore, effective treatments remain crucial to controlling it. As interferon-alpha (IFN-α) and -beta (β) have been proposed as COVID-19 treatments, we sought to assess their effectiveness on respiratory, cardiovascular, neurological, and psychiatric signs and symptoms, as well as PASC and death, in hospitalized COVID-19 patients without multiple sclerosis (MS). Using a federated data research network (TriNetX), we performed a retrospective cohort study of hospitalized COVID-19 patients without MS who received IFN-α or -β treatment, comparing them to a similar cohort who did not receive treatment. Following propensity-score matched analyses, we demonstrate that hospitalized COVID-19 patients who were treated with IFN-α or -β had significantly higher odds of death. In contrast, there was no significant difference in any other outcomes between 1–30 days or 1 day to anytime afterward. Overall, hospitalized COVID-19 patients without MS who were treated with IFN-α or -β had similar short- and long-term sequelae (except for mortality) as those who did not receive treatment. The potential benefits of utilizing IFN-α or -β treatment as therapeutics remain to be realized, and our research highlights the need to explore repurposing drugs for COVID-19 using real-world evidence.

## 1. Introduction

The coronavirus disease 2019 (COVID-19) pandemic has infected more than 704.7 million people and caused more than 7.0 million deaths as of 13 April 2024 [1]. Currently, the United States Food and Drug Administration (FDA) has approved four COVID-19 treatments: Veklury (remdesivir), Olumiant (baricitinib), Actemra (tocilizumab), and Paxlovid (nirmatrelvir and ritonavir) [2]. However, SARS-CoV-2, the causative agent of COVID-19, has continuously mutated, leading to variants such as Omicron, which may escape treatments [3]. For example, monoclonal antibodies, which were previously authorized as COVID-19 treatments, had their approval revoked by the FDA following the emergence of Omicron [4]. In addition, post-acute sequelae of SARS-CoV-2 (PASC), also known as Long COVID, remains an issue for at least 65 million people, yet no treatment has been discovered [5,6]. Therefore, increasing the range of therapeutic options is critical as SARS-CoV-2 evolves and continues to cause COVID-19 and PASC.

Host-directed antivirals, which target host proteins and pathways, are a potent strategy for COVID-19 treatments since they have broad-spectrum activity and decreased viral evasion [7]. One example is interferons (IFN), which are vital components of the innate immune response that respond to viral infection [8]. IFN act to reduce viral levels by inducing antiviral proteins and enhancing the response of immune cells [9].

IFN-alpha (α) and -beta (β) are Type I IFN that have previously received approval from the FDA to treat various conditions [10]. IFN-α has been used for hepatitis B and C, melanomas and other cancers, and genital warts [11]. IFN-β has previously been approved as a treatment for patients with multiple sclerosis (MS) to delay progression of disease [12]. However, IFN-α and -β treatment can lead to side effects such as flu-like symptoms, depression, cognitive and neurological impairments, cardiotoxicity, and pulmonary toxicity [13].

Type I IFN have previously been evaluated for drug repurposing against COVID-19. COVID-19 patients treated with IFN-α, individually or in combination with other therapeutics, had generally improved outcomes [14,15,16]. However, clinical trials evaluating treatments with IFN-β, either alone or in combination with other antivirals, yielded mixed results for COVID-19 patients, ranging from worsened to improved outcomes [17,18,19,20,21,22]. With the lack of conclusive efficacy, in December 2023, the National Institutes of Health (NIH) recommended against using Type I IFN as COVID-19 treatments except in clinical trials [10].

However, few published studies have analyzed real-world evidence of Type I IFN treatment in COVID-19 patients with various disparate outcomes. Therefore, this study aims to determine the effect of IFN-α and -β treatment on respiratory, cardiovascular, neurological, and psychiatric outcomes, PASC, and death in hospitalized COVID-19 patients. Through our work, we will contribute toward identifying whether IFN-α and -β may serve as effective therapeutics against COVID-19. 

## 2. Materials and Methods

### 2.1. Database Network

This study was conducted with data obtained from TriNetX, LLC (TriNetX), a global federated health research network that furnishes access to electronic medical records (EMRs) from more than 300 million patients. TriNetX is continuously updated with data from healthcare organizations worldwide and includes demographics, diagnoses, procedures, medications, and labs. These data are de-identified, exempting them from Institutional Review Board approval. TriNetX complies with the United States’ Health Insurance Portability and Accountability Act (HIPAA) and the European Union’s General Data Protection Regulation (GDPR).

### 2.2. Study and Cohort Design

**Figure 1:**
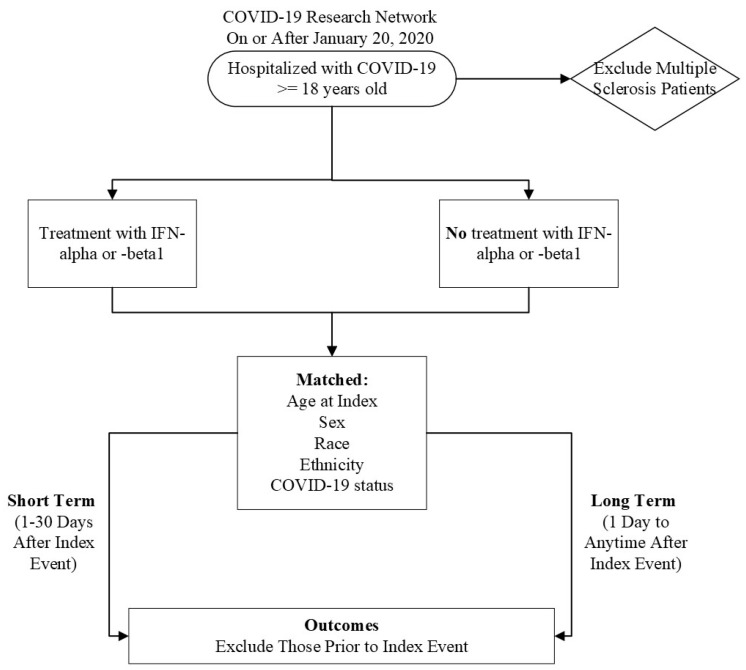
Flow chart of experimental design.

Initially, we characterized our cohort of interest using the TriNetX Research Network. We then performed a retrospective cohort study using the COVID-19 Research Network (Figure 1). Our study time frame began on 20 January 2020, when COVID-19 was officially diagnosed in the United States of America, until the date of analysis. Our exposed population was patients who were ≥18 years old and had a diagnosis of COVID-19 but did not have MS. A patient was considered to have COVID-19 if they had the ICD-10-CM code U07.1 or had a positive result, as compiled and aggregated by TriNetX. A complete list of codes is included in Table S1. These patients received IFN-α or -β treatment after their initial COVID-19 diagnosis. Altogether, these criteria were defined as our “index event”, or the onset of disease and the identified conditions. 

Our unexposed, or comparison, cohort were COVID-19 patients who were ≥18 years old without multiple sclerosis who did not receive IFN-α or -β treatment after diagnosis. MS patients were excluded because IFN-β is an approved therapy for this autoimmune and chronic disease, creating a likelihood that MS patients may have already been treated with IFN-β prior to contracting COVID-19, which could confound the results [23].

To account for potential confounders, the cohorts were propensity-score matched (1:1) within TriNetX [24]. Specifically, propensity-score matching was performed using a logistic regression model and greedy nearest neighbor technique with a caliper of 0.1 pooled standard deviations. The following variables were matched: Age at index, Race, Ethnicity, Sex, and COVID-19 status. All statistical analyses were performed using TriNetX [24]. We utilized odds ratios and 95% confidence intervals to determine the odds of developing an outcome. If a patient had an outcome before the index event, they were excluded from calculations.

### 2.3. Outcomes

The respiratory outcomes of interest were assistance with respiratory ventilation at high nasal flow/velocity (ICD-10-PCS: 5A0935A, 5A0945A, and 5A0955A), dependence on respiration (ventilator) (ICD-10-CM: Z99.11), pneumonia (ICD-10-CM: J12.82, J18, and J18.9), dyspnea (ICD-10-CM: R06.00 and R06.02), hypoxemia (ICD-10-CM: R09.02), acute respiratory distress syndrome (ICD-10-CM: J80), acute respiratory failure (ICD-10-CM: J96.0 and J96.00), respiratory ventilation (ICD-10-PCS: 5A1935Z, 5A1945Z, and 5A1955Z), PASC (ICD-10-CM: U09), and death (deceased). These have previously been associated with COVID-19 and are indicators of disease severity [20].

The cardiovascular outcomes of interest were essential (primary) hypertension (ICD-10-CM: I10), atrial fibrillation and flutter (ICD-10-CM: I48), acute pericarditis (ICD-10-CM: I30), heart failure (ICD-10-CM: I50), acute myocardial infarction (ICD-10-CM: I21), cardiac arrest (ICD-10-CM: I46), and pulmonary embolism (ICD-10-CM: I26). These outcomes have been identified as being elevated in COVID-19 patients [25]. 

The neurological and psychiatric outcomes of interest were nerve, nerve root, and plexus disorders (ICD-10-CM: G50-G59), diseases of myoneural junction and muscle (ICD-10-CM: G70-G73), dementia (ICD-10-CM: F01 or F02), substance use disorders (ICD-10-CM: F10-F19), psychotic, mood, and anxiety disorders (ICD-10-CM: F20-F48), and insomnia (ICD-10-CM: F51 or G47.0) [26].

## 3. Results

### 3.1. Cohort Characteristics: Research Network

To first characterize hospitalized COVID-19 patients who received Type I IFN treatment, we utilized the TriNetX Research Network. As of 8 October 2023, this network contained data from 111,128,059 patients from 78 HCOs in 4 different countries. The characterization of this data was performed on 8 October 2023, and we had a total of 48 patients who fit the inclusion criteria. 

There was an eqaul division of males and females. In the cohort, 64% were non-Hispanic or Latino, while 20% were Hispanic or Latino. Most of the cohort was White (62%), while 20% were Black or African American. These patients were generally unhealthier before contracting COVID-19 and receiving Type I IFN treatment. Approximately 1–30 days prior, 44% of the cohort had circulatory system diseases, and 42% had neoplasms or endocrine, nutritional, and metabolic diseases. Interestingly, 29% of the patients had been previously diagnosed with COVID-19. About 29% of the cohort had diseases of the nervous or respiratory system, while a quarter had acute kidney failure and chronic kidney disease. 

In terms of medications, 63% of the cohort were using central nervous system medications, with 50% being given analgesics. In addition, 60% were using cardiovascular medications. Interestingly, 58% were using antimicrobials, and 35% were using penicillin and beta-lactam antimicrobials. Related to diabetes, 27% of the cohort used blood glucose regulation agents. 

### 3.2. Outcomes: Research Network

To briefly understand the outcomes experienced by this cohort, we examined the percentage of cohort members who developed pneumonia, PASC, or died. When we reviewed outcomes 1–30 days afterward, 96.2% of the cohort had not developed pneumonia, and 91.3% survived. Because PASC is defined as having signs, symptoms, and conditions that are present or develop four or more weeks after SARS-CoV-2 infection, our time frame was one day until 8 October 2023 [5]. We saw that 96.4% of the cohort did not develop PASC. Therefore, these patients generally had positive outcomes after hospitalization, COVID-19 infection, and treatment with a Type I IFN.

### 3.3. Cohort Characteristics: COVID-19 Research Network

Based on our results from the Research Network, we then decided to utilize the TriNetX COVID-19 Research Network, which includes a subset of HCOs that previously indicated a desire to contribute toward COVID-19 research [27]. It included data from 111,663,882 patients from 87 HCOs in 12 different countries. The analysis of this data was performed on 27 October 2023.

There was a total of 238 patients who were ≥18 years old without MS and were treated with a Type I IFN after their first instance of COVID-19 (Table 1). 

More than half of the patients were male (62%), with an average age of 61.3 years at the time of analysis. However, 66% of the patients’ race and 64% of their ethnicity were unknown. More than 74% of the patients had already been diagnosed with COVID-19. One month prior to meeting the inclusion criteria, 4% of the cohort was diagnosed with hepatitis B, and 4% of the cohort had hepatitis C. Approximately 4% of the cohort was diagnosed with non-Hodgkin lymphoma, and 4% had malignant melanomas. No one had been diagnosed with hairy cell leukemia, Kaposi’s sarcoma, or genital warts.

Patients in our cohorts were also receiving other medications in addition to Type I IFN. Antivirals were utilized by 48% of the cohort of interest. In addition, 46% of them were using respiratory tract medications alongside Type I IFN. Hydroxychloroquine, which was eventually disproven as a therapy for COVID-19, was used by 42% of the cohort [28]. Approximately 11% of the cohort of interest was treated with immunological agents as well.

In our comparison cohort, there was a total of 1,041,910 patients who met the criteria. However, when looking at baseline characteristics, information was only available for 909,529 patients (Table S2). The average age of the patients was 55.7 years old, and 42% of the patient population were males. Approximately 68% of the patients were non-Hispanic or Latino, and 63% were White. Only 9% of the cohort had been infected with COVID-19 beforehand. A total of 306 patients (0.03%) of the cohort had hepatitis B, and 1,175 (0.13%) of the cohort had hepatitis C. Only 340 patients (0.04%) in the cohort were diagnosed with non-Hodgkin lymphoma, 572 (0.06%) had malignant melanoma, 25 (0.002%) had hairy cell leukemia, 26 (0.003%) had been diagnosed with Kaposi’s sarcoma, and 174 (0.02%) had genital warts. 

In contrast to our cohort of interest, only 3% of the comparison cohort was using antivirals, though 20% were using respiratory tract medications such as bronchodilators. Only 1788 patients, or 0.20% of our comparison cohort, were using hydroxychloroquine, and 4% were treated with immunological agents. Thus, the comparison cohort appeared to be taking less medications than the cohort of interest, which may suggest that they were relatively healthier.

After propensity-score matching, there were a total of 231 patients in each cohort. Their characteristics were relatively similar, as shown in Table 2. The average age was 61.4 years in our cohort of interest, while it was 61.5 years in our comparison cohort. Approximately 61.9% of our cohort of interest was male, which was nearly the same percentage of males in the comparison cohort (60.6%). In terms of ethnicity, 31.2% of the cohort treated with Type I IFN was non-Hispanic or Latino, while 29.4% of the comparison cohort was non-Hispanic or Latino. Both cohorts were composed of 26.4% Whites. There was slightly more Blacks or African Americans in the cohort of interest (4.8%) compared to the comparison cohort (4.3%). In both cohorts, approximately 71.9% of the patients were diagnosed with COVID-19.

### 3.4. Respiratory Outcomes and Death 1–30 Days Afterwards: COVID-19 Research Network

**Figure 2:**
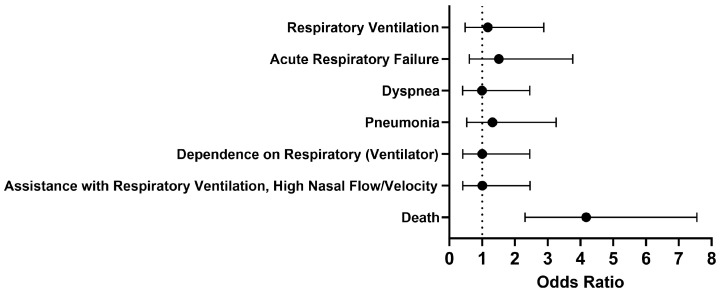
Odds ratio of short-term respiratory outcomes and mortality in COVID-19 patients with Type I IFN compared to COVID-19 patients without Type I IFN treatment, 1-30 days afterwards.

Overall, no significant differences were seen in respiratory outcomes in hospitalized COVID-19 patients without MS who were treated with a Type I IFN compared to those who were untreated (Figure 2). The odds of the examined outcomes were about the same or slightly higher in the cohort of interest compared to our comparison cohort. There was no significant difference between the cohorts 1 to 30 days after treatment in terms of respiratory ventilation (OR [95% CI]: 1.2 [0.5–2.9]), acute respiratory failure (OR [95% CI]: 1.5 [0.6–3.8]), dyspnea (OR [95% CI]: 1.0 [0.4–2.5]), pneumonia (OR [95% CI]: 1.3 [0.5–3.3]), dependence on the respirator (ventilator) (OR [95% CI]: 1.0 [0.4–2.5]), and assistance with respiratory ventilation at high nasal flow/velocity (OR [95% CI]: 1.0 [0.4–2.5]). However, the odds of death were 4.2 times higher in COVID-19 patients without MS who received Type I IFN treatment compared to those who did not. When looking more closely at the cohort treated with IFN-α or -β, the survival probability was 76.0%, with 173 out of 227 patients surviving. 

### 3.5. Respiratory Outcomes, Death, and PASC 1 Day Afterwards—27 October 2023: COVID-19 Research Network

**Figure 3:**
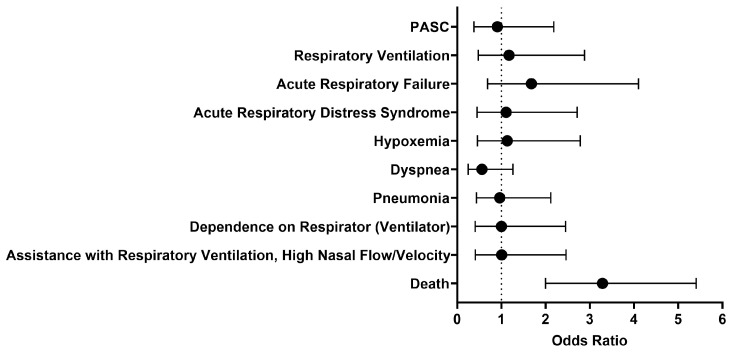
Odds ratio of respiratory outcomes, PASC, and mortality in COVID-19 patients with Type I IFN treatment compared to COVID-19 patients without Type I IFN treatment, 1 day to anytime afterwards.

We then looked at outcomes one day to anytime afterwards, and the analysis was performed on October 27, 2023. The findings were generally similar to what was observed between 1 to 30 days after initial treatment with either IFN-α or -β (Figure 3). The odds of respiratory ventilation (OR [95% CI]: 1.2 [0.5–2.9]), acute respiratory failure (OR [95% CI]: 1.7 [0.7–4.1]), acute respiratory distress syndrome (OR [95% CI]: 1.1 [0.5–2.7]), hypoxemia (OR [95% CI]: 1.1 [0.5–2.8]), developing pneumonia (OR [95% CI]: 1.0 [0.4–2.1]), depending on a respirator (ventilator) (OR [95% CI]: 1.0 [0.4–2.5]), and needing assistance with respiratory ventilation at high nasal flow/velocity (OR [95% CI]: 1.0 [0.4–2.5]) were all approximately the same. The cohort treated with IFN-α or -β had slightly lower odds of being diagnosed with dyspnea (OR [95% CI]: 0.6 [0.2–1.3]) and PASC (OR [95% CI]: 0.9 [0.4–2.2]). Though there was generally no significant difference in selected outcomes between the two cohorts, the cohort that received Type I IFN treatment had 3.3 times higher odds of dying as compared to the comparison cohort. However, the survival probability of the treated cohort was 70.4%, as 160 out of 227 patients survived. 

### 3.6. Cardiovascular Outcomes 1 Day to Anytime Afterwards: COVID-19 Research Network

Since SARS-CoV-2 not only affects the respiratory tract, we decided to explore other systems such as the cardiovascular system. COVID-19 has previously been shown to increase the risk of cardiovascular issues such as heart attack and stroke up to a year post infection [25]. Therefore, we decided to investigate whether treatment with Type I IFN would affect cardiovascular outcomes. The analysis was performed on 10 March 2024, and there were 275 patients in each cohort.

**Figure 4:**
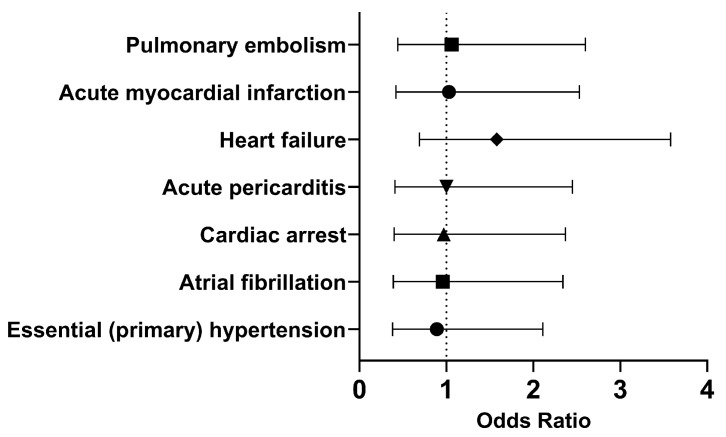
Odds ratio of cardiovascular outcomes in COVID-19 patients with Type I IFN treatment compared to COVID-19 patients without Type I IFN treatment, 1 day to anytime afterwards.

In both cohorts, most of the patients did not develop the selected outcomes within 30 days. Therefore, we investigated the long-term outcomes (Figure 4). The odds of having essential (primary) hypertension (OR [95% CI]: 0.89 [0.38, 2.11]), atrial fibrillation (OR [95% CI]: 0.96 [0.39, 2.34]), or cardiac arrest (OR [95% CI]: 0.97 [0.40, 2.37]) were slightly lower in the cohort of interest compared to the comparison cohort. However, COVID-19 patients who had been treated with Type I IFN had approximately the same or slightly higher odds of developing acute pericarditis (OR [95% CI]: 1.00 [0.41, 2.45]), heart failure (OR [95% CI]: 1.58 [0.69, 3.58]), acute myocardial infarction (OR [95% CI]: 1.03 [0.42, 2.53]), and pulmonary embolism (OR [95% CI]: 1.06 [0.44, 2.60]). Overall, it appears that the odds of developing cardiovascular issues were approximately the same in both cohorts in the long term.

### 3.7. Neurological and Psychiatric Outcomes 1 Day to Anytime Afterwards: COVID-19 Research Network

Lastly, using the previously described criteria, we decided to explore neurological and psychiatric sequelae in our cohorts. A side effect of Type I IFN treatment includes development or worsening of mental illness [11,12]. In addition, six months following SARS-CoV-2 infection, neurological and psychiatric sequelae may occur, though these outcomes may be transient [26,29]. Therefore, it was important to understand what neurological and psychiatric outcomes patients treated with Type I IFN might experience. The analysis was performed on March 10, 2024, and each cohort had 275 patients following propensity-score matching.

Within thirty days of hospitalization with COVID-19 and Type I IFN treatment, the cohort of interest had approximately the same odds as the comparison cohort of having nerve, nerve root, and plexus disorders (OR [95% CI]: 0.98 [0.40, 2.40]), substance use disorders (OR [95% CI]: 1.01 [0.41, 2.48]), psychotic, mood, and anxiety disorders (OR [95% CI]: 0.94 [0.38, 2.30]), and insomnia (OR [95% CI]: 1.05 [0.43, 2.57]). Therefore, in the short term, using Type I IFN neither alleviated nor increased the odds of these neurological and psychiatric disorders. 

**Figure 5:**
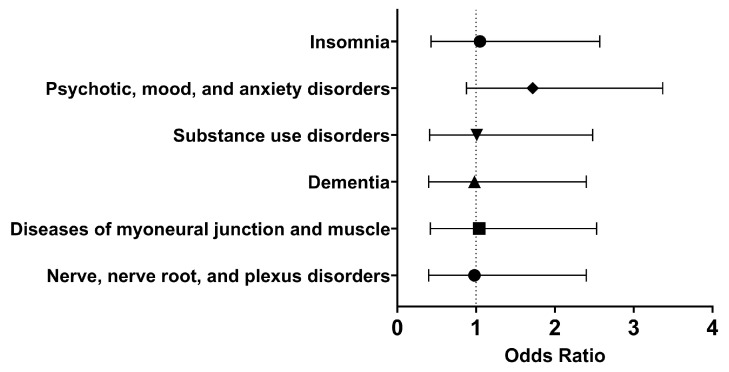
Odds ratio of neurological and psychiatric outcomes in COVID-19 patients with Type I IFN treatment compared to COVID-19 patients without Type I IFN treatment, 1 day to anytime afterwards.

In the long term, there were similar odds of developing nerve, nerve root, and plexus disorders (OR [95% CI]: 0.98 [0.40, 2.40]), diseases of myoneural junction and muscle (OR [95% CI]: 1.04 [0.42, 2.53]), dementia (OR [95% CI]: 0.98, 0.40, 2.40), substance use disorders (OR [95% CI]: 1.01 [0.41, 2.48]), psychotic, mood, and anxiety disorders (OR [95% CI]: 1.72 [0.88, 3.37]), and insomnia (OR [95% CI]: 1.05 [0.43, 2.57]) (Figure 5). Therefore, it appeared that Type I IFN treatment did not significantly affect selected neurological and psychiatric sequelae.

## 4. Discussion

Overall, hospitalized COVID-19 patients without MS who were treated with Type I IFN had higher odds of death but similar odds of selected respiratory, cardiovascular, neurological, and psychiatric outcomes. Though side effects have been reported following IFN treatment, based on our results, no major differences were seen in the short and long term. In terms of neurological and psychiatric sequelae, this is important because Type I IFN treatment can lead to severe side effects, such as depression, mood and behavior problems, and suicidal ideation [11,12]. Therefore, our study suggests that Type I IFN treatment may not significantly affect long-term neurological and psychiatric outcomes. 

The most significant difference between cohorts in the short and long term was mortality. In the short term, the odds of mortality is 4.2 times greater in Type I IFN-treated patients compared to untreated hospitalized patients. The odds of mortality decreases from 4.2 (1–30 days after treatment) to 3.3 (1 day to anytime afterwards), suggesting that death occurred soon after treatment was initiated. Though the odds of mortality are higher in our treated cohort in the short and long term, the survival probability was 69.4% and 76.0%, respectively. However, the cause of death may not be related to the respiratory, cardiovascular, neurological, and psychiatric sequelae we examined. Therefore, our next steps would be to further determine what may have contributed to mortality.

An important consideration is that patients in our cohort of interest were generally unhealthier than our comparison cohort. When looking at the Research Network, nearly a third or half of the patients had pre-existing health conditions and were taking medications. In the COVID-19 Research Network, nearly 75% of the cohort had already been diagnosed with COVID-19. Our cohort was also composed of older adults (average age: 61.2 years), so they may have been at higher risk of severe disease [30]. Thus, IFN treatment may not have been as effective in this cohort as it might be in other populations. This highlights the importance of patient selection for IFN therapy.

In addition, treatment with Type I IFN may not directly affect the respiratory, cardiovascular, or neurological systems. IFN-α was given either subcutaneously, intramuscularly, intravenously, or intralesionally, while IFN-β was given subcutaneously [11,12]. Mechanistically, Type I IFN induce an antiviral state in the host by activating the innate immune response. Hundreds of antiviral genes restrict viral infection by targeting the viral life cycle, suppressing viral replication, enhancing detection of pathogens, and further signaling of the innate immune response [31]. This resistance to viral infection is nonspecific though [32,33]. Timing is also important in IFN production, as SARS-CoV-2 is able to delay the Type I IFN response. As the IFN response may have already been induced by the time the COVID-19 patient was hospitalized and treated with Type I IFN, the timing of treatment may have affected our results. If patients were treated with Type I IFN prior to infection, this may prepare the immune system to defend itself against SARS-CoV-2 infection, leading to a quicker immunological response and improved outcomes. In preclinical studies, early Type I IFN treatment led to decreased viral titers and limited weight loss [34,35]. Therefore, the timing of IFN therapy is a crucial factor that must be considered to optimize benefits while minimizing harm.

Another point is whether SARS-CoV-2 variants of concern affected the effectiveness of IFN treatment. In cell culture models, SARS-CoV-2 variants of concern appeared to have greater resistance to IFN treatment [36]. Although it is not possible to know exactly which variant patients were infected with, as these data were not available, we could potentially correspond time of infection with the variant circulating in the future. Briefly, we saw that the greatest number of patients were diagnosed with COVID-19 in 2020 and 2022. During these years, the original SARS-CoV-2 and the SARS-CoV-2 Omicron variant were spreading worldwide.

A potential bias is the inability to follow up with participants, as some patients may have experienced outcomes that were not reported. In addition, the patients in both cohorts were hospitalized, which may lead to selection bias. The number of patients experiencing outcomes was often ≤10, which may affect the odds ratios. This also suggests that these outcomes were relatively rare in the cohorts. Though we tried to include as many potential confounders as possible, others may remain unaccounted for. Patients younger than 18 years old were excluded from the study, and as older adults are at higher risk of severe disease, this may have affected our outcomes. However, we attempted to account for this important risk factor through propensity-score matching.

Our work lends support to the NIH’s position on Type I IFN as treatment options for COVID-19. However, patients treated with Type I IFN were generally unhealthier than the comparison cohort, meaning that these results should be taken with caution. These findings may not apply to patients with MS, as they were excluded from this comparison. The patients in our cohort were also derived from multiple countries, so there might be different treatment strategies and other underlying factors that cannot be reflected in the data. We also emphasize that Type I IFN treatment may have been given individually or in combination with other medications. Generally, though, Type I IFN did not appear to protect against mortality, though we observed similar odds of morbidity in the short and long term.

A potential future direction is to examine whether patients who received Type I IFN treatment prior to diagnosis with COVID-19 had improved outcomes, as earlier treatment with Type I IFN may improve outcomes. In addition, it would be interesting to include patients with MS and see if the results differed than what was found here. Another possibility would be to select different outcomes related to the gastrointestinal tract, kidneys, or reproductive tract, as SARS-CoV-2 has demonstrated the ability to affect these systems as well [5]. Lastly, we are interested in utilizing causal inference to determine the effectiveness of Type I IFN amongst the hospitalized COVID-19 patients who received treatment.

As COVID-19 continues to plague our society, discovering effective therapeutics against SARS-CoV-2 and other coronaviruses remains a priority. Though Type I IFN may not be a recommended treatment option for COVID-19, our work has broader underlying implications. As our study demonstrates, it is difficult to identify novel treatments for COVID-19. Traditionally, it takes 10 years and between 1.2–2.5 billion dollars for a drug to reach the clinic [37]. However, the COVID-19 pandemic, the mpox (formerly known as monkeypox) outbreak, and other pathogens have demonstrated the ability to emerge and rapidly spread worldwide. Thus, we need to find treatments against potential pandemic pathogens to prevent future pandemics.

## 5. Conclusions

Our retrospective cohort study revealed that hospitalized COVID-19 patients treated with Type I IFN had higher odds of death in the short and long term, compared to hospitalized COVID-19 patients who were not treated with Type I IFN. However, both cohorts experienced similar odds of respiratory, cardiovascular, neurological, and psychiatric sequelae. It is important to note that the cohort given Type I IFN was generally unhealthier, which may influence the outcomes. Our research highlights the importance of using real-world evidence to investigate and repurpose treatments against potential pandemic pathogens. 

## Figures and Tables

**Table 1:** Baseline characteristics of cohort of interest in the COVID-19 Research Network.

Category	Sub-Category	Patients, n = 238 (%)
Sex	Male	148 (62%)
	Female	78 (33%)
	Unknown	12 (5%)
Ethnicity	Not Hispanic or Latino	72 (30%)
	Hispanic or Latino	10* (4%)
	Unknown	159 (66%)
Race	White	65 (27%)
	Black or African American	11 (4%)
	Asian	10* (4%)
	Native Hawaiian or other	10* (4%)
	Other	10* (4%)
	Unknown	154 (64%)
Age at index (years)	Mean	61.2
	Standard deviation	15.5
	Minimum	18
	Maximum	87
COVID-19 diagnosis		173 (73%)
Overweight, obesity, and other hyperalimentation		31 (13%)
Essential (primary) hypertension		82 (34%)
Diabetes mellitus		39 (16%)
Chronic lower respiratory diseases		35 (15%)
	Other chronic obstructive pulmonary disease	16 (7%)
Nicotine dependence		14 (6%)
Heart diseases	Ischemic heart diseases	19 (8%)
	Other forms of heart disease	47 (20%)
Acute kidney failure and chronic kidney disease		60 (25%)
	Chronic kidney disease	34 (14%)
Diseases of liver		16 (7%)
Neoplasms		49 (21%)
	Malignant neoplasms of lymphoid, hematopoietic, and related tissue	22 (9%)
Certain disorders involving the immune mechanism		24 (10%)
Antivirals		114 (48%)
	Lopinavir	88 (37%)
	Ritonavir	88 (37%)
Respiratory tract medications		109 (46%)
Hydroxychloroquine		99 (42%)
Immunological agents		25 (11%)

* To protect patient privacy, the minimum number of patients shown in TriNetX is 10.

**Table 2:** Characteristics of cohorts after propensity-score matching.

Category	With Treatment n (%)	Without Treatment n (%)
Total Patients	231	231
Average Age at Index	61.4 years	61.5 years
Male	143 (61.9%)	140 (60.6%)
White	61 (26.4%)	61 (26.4%)
Black or African American	11 (4.8%)	10 (4.3%)
Not Hispanic or Latino	72 (31.2%)	68 (29.4%)

To protect patient privacy, the minimum number of patients shown in TriNetX is 10.

## Data Availability

Data are available on the TriNetX Analytics Network (https://trinetx.com).

## Supplementary Materials

The following supporting information can be downloaded at: https://www.mdpi.com/article/10.3390/pathogens13070539/s1, Table S1: Code for creation of cohorts; Table S2: Baseline characteristics of the cohort of interest in the COVID-19 Research Network.
