# Supplementary material for: Evaluation of Type I Interferon Treatment in Hospitalized COVID-19 Patients: A Retrospective Cohort Study
Source: Pathogens. 2024 Jun 26;13(7):539. doi: 10.3390/pathogens13070539 (PMC11280121; doi:10.3390/pathogens13070539)
Supplement: Supplementary file 1 [file pathogens-13-00539-s001.zip › pathogens-3047497-supplementary.pdf]

Supplementary Table S1. Codes for creation of cohorts.

Category	Code	Description	Filter
Hospitalization		Visit: Inpatient Encounter	
	HCPCS 60378	Hospital observation service, per hour	
	HCPCS G0379	Direct admission of patient for hospital observation care	
	CPT 99221	Initial hospital care, per day, for the evaluation and management of a patient, which requires these 3 key components: A detailed or comprehensive history; A detailed or comprehensive examination; and Medical decision making that is straightforward or of low complexity. Counseling and/or coordination of care with other physicians, other qualified health care professionals, or agencies are provided consistent with the nature of the problem(s) and the patient's and/or family's needs. Usually, the problem(s) requiring admission are of low severity. Typically, 30 minutes are spent at the bedside and on the patient's hospital floor or unit.	
	CPT 99222	Initial hospital care, per day, for the evaluation and management of a patient, which requires these 3 key components: A comprehensive history; A comprehensive examination; and Medical decision making of moderate complexity. Counseling and/or coordination of care with other physicians, other qualified health care professionals, or agencies are provided consistent with the nature of the problem(s) and the patient's and/or family's needs. Usually, the problem(s) requiring admission are of moderate severity. Typically, 50 minutes are spent at the bedside and on the patient's hospital floor or unit.	
	CPT 99223	Initial hospital care, per day, for the evaluation and management of a patient, which requires these 3 key components: A comprehensive history; A comprehensive examination; and Medical decision making of high complexity. Counseling and/or coordination of care with other physicians, other qualified health care professionals, or agencies are provided consistent with the nature of the problem(s) and the patient's and/or family's needs. Usually, the problem(s) requiring admission are of high severity. Typically, 70 minutes are spent at the bedside and on the patient's hospital floor or unit.	
SARS-CoV-2	TNX Curated 9088	SARS coronavirus 2 and related RNA [Presence]	≥ 18 years AND Positive
	ICD-10-CM U07.1	COVID-19	≥ 18 years
Multiple sclerosis	ICD-10-CM G35	Multiple sclerosis	
Treatment	RxNorm 75917	interferon beta-1a	
	RxNorm 72257	interferon beta-1b	
	LOINC 59181-8	Interferon beta (given) [Type]	
	HCPCS J1826	Injection, interferon beta-1a, 30 mcg	
	HCPCS Q3027	Injection, interferon beta-1a, 1 mcg for intramuscular use	
	HCPCS Q3028	Injection, interferon beta-1a, 1 mcg for subcutaneous use	
	HCPCS J1830	Infection, interferon beta-1b, 0.25 mg (code may be used for medicare when drug administered under the direct supervision of a physician, not for use when a drug is self administered)	
	RxNorm 1546168	peginterferon beta-1a	
	HCPCS J1830	Injection, interferon beta-1b, 0.25 mg (code may be used for medicare when drug administered under the direct supervision of a physician, not for use when drug is self administered)	
Treatment	RxNorm 120608	peginterferon alfa-2a	
	RxNorm 2587059	ropeginterferon alfa-2b	
	RxNorm 5880	interferon alfa-2b	
	RxNorm 612937	interferon alfa-n3	
	HCPCS J9213	Injection, interferon, alfa-2a, recombinant, 3 million units	
	HCPCS J9214	Injection, interferon, alfa-2b, recombinant, 1 million units	
	HCPCS J9215	Injection, interferon, alfa-n3, (human leukocyte derived), 250,000 iu	
	HCPCS S0145	Injection, pegylated interferon alfa-2a, 180 mcg per ml	
	HCPCS S0148	Injection, pegylated interferon alfa-2b, 10 mcg	

Supplementary Table S2. Baseline characteristics of the cohort of interest in the COVID-19 Research Network.

Category	Sub-category	ICD-10-CM Code	Patients, n = 238 (Percentage)
Sex	Male		148 (62%)
	Female		78 (33%)
	Unknown		12 (5%)
Ethnicity	Not Hispanic or Latino		72 (30%)
	Hispanic or Latino		10* (4%)
	Unknown		159 (66%)
Race	White		65 (27%)
	Black or African American		11 (4%)
	Asian		10* (4%)
	Native Hawaiian or Other		10* (4%)
	Other		10* (4%)
	Unknown		154 (64%)
Age at Index (Years)	Mean		61.2
	Standard Deviation		15.5
	Minimum		18
	Maximum		87
COVID-19 Diagnosis		U07.1	173 (73%)
Overweight, obesity, and other hyperalimentation		E65-E68	14 (6%)
Essential (primary) hypertension		I10	19 (8%)
Diabetes mellitus		E08-E13	47 (20%)
Chronic lower respiratory diseases		J40-J47	60 (25%)
	Other chronic obstructive pulmonary disease	J44	34 (14%)
Nicotine dependence		F17	16 (7%)
Heart diseases	Ischemic heart diseases	I20-I25	49 (21%)
	Other forms of heart disease	I30-I5A	22 (9%)
Acute kidney failure and chronic kidney disease		N17-N19	24 (10%)
	Chronic kidney disease	N18	114 (48%)
Diseases of liver		K70-K77	88 (37%)
Neoplasms		C00-D49	88 (37%)
	Malignant neoplasms of lymphoid, hematopoietic, and related tissue	C81-C96	109 (46%)
Certain disorders involving the immune mechanism		D80-D89	99 (42%)
Antivirals		AM000	25 (11%)
	Lopinavir	195088	
	Ritonavir	85762	
Respiratory Tract Medications		RE000	
Hydroxychloroquine		5521	
Immunological agents		IM000	
